# Supplementary material for: Global hypo-methylation in a proportion of glioblastoma enriched for an astrocytic signature is associated with increased invasion and altered immune landscape
Source: eLife. 2022 Nov 22;11:e77335. doi: 10.7554/eLife.77335 (PMC9681209; doi:10.7554/eLife.77335)
Supplement: Figure 2—source data 1. [file elife-77335-fig2-data1.zip › Figure_2_source_data_1/Figure_2I_J/homerResults/motif6.similar.html]

motif6

## Information for motif6

A
G
T
C
G
C
A
T
T
A
C
G
G
C
A
T
A
C
T
G
A
G
T
C
G
A
C
T
G
T
A
C
C
T
G
A
A
C
T
G
G
A
T
C
T
A
G
C
T
A
G
C
G
C
A
T
T
A
C
G
  
Reverse Opposite:  

A
T
G
C
C
G
T
A
A
T
C
G
A
T
C
G
C
T
A
G
G
T
A
C
G
A
C
T
A
C
T
G
C
T
G
A
T
C
A
G
T
G
A
C
C
G
T
A
A
T
G
C
C
G
T
A
T
C
A
G
  

|  |  |
| --- | --- |
| p-value: | 1e-47 |
| log p-value: | -1.083e+02 |
| Information Content per bp: | 1.641 |
| Number of Target Sequences with motif | 51.0 |
| Percentage of Target Sequences with motif | 7.02% |
| Number of Background Sequences with motif | 1.1 |
| Percentage of Background Sequences with motif | 0.40% |
| Average Position of motif in Targets | 117.4 +/- 41.9bp |
| Average Position of motif in Background | 81.2 +/- 10.6bp |
| Strand Bias (log2 ratio + to - strand density) | 0.1 |
| Multiplicity (# of sites on avg that occur together) | 1.05 |
| Motif File: | file (matrix) reverse opposite |

### Similar de novo motifs found

|  |  |  |  |  |  |  |  |
| --- | --- | --- | --- | --- | --- | --- | --- |
| Rank | Match Score | Redundant Motif | P-value | log P-value | % of Targets | % of Background | Motif file |
| 1 | 0.633 | T G C A A T G C C G T A A C T G A T C G A C T G A T G C G C T A T A G C C G T A A T C G C T G A T C A G T A G C G T C A | 1e-31 | -73.168423 | 5.37% | 0.58% | motif file (matrix) |
| 2 | 0.626 | A T C G A T G C G C A T T A G C C G T A A T C G T A G C G T A C C G T A C A T G A T C G A C G T | 1e-28 | -64.941444 | 4.96% | 0.39% | motif file (matrix) |
| 3 | 0.667 | A G T C C G A T A T C G C T G A T C A G T C G A T A C G T A G C C G T A T A C G | 1e-27 | -63.755690 | 6.34% | 1.04% | motif file (matrix) |
| 4 | 0.607 | A G T C C G T A A T C G A C T G A C T G A G T C | 1e-16 | -38.793178 | 39.94% | 25.75% | motif file (matrix) |
